# Caffeinated Chewing Gum Improves Basketball Shooting Accuracy and Physical Performance Indicators of Trained Basketball Players: A Double-Blind Crossover Trial

**DOI:** 10.3390/nu16091256

**Published:** 2024-04-24

**Authors:** Hou-Shao Liu, Chi-Chu Liu, Yi-Jie Shiu, Pei-Tzu Lan, An-Yu Wang, Chih-Hui Chiu

**Affiliations:** 1 Institute of Sports Sciences, University of Taipei, Taipei 111036, Taiwan; 4im1030027@gmail.com; 2 Department of Ball Sport, National Taiwan University of Sport, Taichung 404, Taiwan; chichu@ntus.edu.tw; 3 Department of Physical Education and Sport Sciences, National Taiwan Normal University, Taipei 106, Taiwan; shiu880511@gmail.com; 4 Department of Bioinformatics and Medical Engineering, Asia University, Taichung 404, Taiwan; 112225001@live.asia.edu.tw; 5 Student Affairs Office, National Taichung University of Science and Technology, Taichung 403, Taiwan; giting1126@gmail.com; 6 Department of Exercise Health Science, National Taiwan University of Sport, Taichung 404, Taiwan

**Keywords:** fatigue index, ergogenic aids, exercise performance

## Abstract

(1) Background: This study investigated the effects of caffeinated chewing gum on the basketball-specific performance of trained basketball players. A double-blind, randomized crossover design was employed. (2) Methods: Fifteen participants (age: 20.9 ± 1.0 years; height: 180.9 ± 5.4 cm; mass: 77.2 ± 7.5 kg; training age: 8.2 ± 0.3 years) were recruited and divided into a caffeine trial (CAF) and placebo trial (PL). The participants in the CAF trial chewed gum containing 3 mg/kg of caffeine for 10 min, while those in the PL trial chewed a placebo gum without caffeine. Following a 15 min rest, all the participants completed basketball-specific performance tests. (3) Results: The free throw accuracy for the CAF trial was significantly higher than that for the PL trial (CAF: 79.0 ± 4.31%; PL: 73.0 ± 9.16%; *p* = 0.012; Cohen’s *d* = 0.94). Additionally, the CAF trial demonstrated significantly better performance in the 20 m segmented dash (CAF: 2.94 ± 1.12 s; PL: 3.13 ± 0.10 s; *p* < 0.001; Cohen’s *d* =1.8) and squats (*p* < 0.05), and exhibited lower fatigue indexes (CAF: 3.6 ± 1.6%; PL: 5.2 ± 1.6%; *p* = 0.009; Cohen’s *d* =1.0). (4) Conclusions: These findings suggest that chewing gum containing 3 mg/kg of caffeine offers moderate-to-large improvements in key performance aspects relevant to professionally trained basketball players.

## 1. Introduction

Basketball is a sport demanding both exceptional physical performance indicators and refined technical skills. Training programs typically focus on enhancing these specialized physical and technical abilities to optimize athletic performance. Basketball drills and games involve frequent high-intensity movements like sprints and jumps, necessitating the development of power-related attributes such as agility, sprinting, and vertical jump height [1,2]. Technical proficiency is also important, encompassing movement skills and the ability to shoot accurately from different positions and distances. Refining these specific skills holds the key to enhancing winning percentages in basketball [3]. In a systematic review article, caffeine supplementation was found to be effective for improving basketball athletes’ vertical jump height, agility, and repeated sprinting ability [4]. These performance indicators are a vital component of enhanced athletic performance among basketball players.

Caffeine (1,3,7-trimethylxanthine) is a popular nutritional supplement among athletes and is readily found in various dietary sources, such as coffee, chocolate, tea, cola, and energy drinks. Research has suggested that caffeine supplementation has a moderate-to-low effect size on strength and sprinting performance [5]. Its mechanism involves antagonizing adenosine receptors to reduce fatigue. In addition, caffeine enhances the ability of the plasma reticulum to release calcium ions, maintains sodium–potassium ATPase (Na^+^/K^+^-ATPase activity), and increases glycolysis [6,7]. Furthermore, caffeine supplementation has been found to benefit limb control and cognitive function [8], both relevant to specialized technical skills in basketball. A pre-exercise caffeine intake of 3–6 mg/kg of body weight has been shown to effectively enhance performance in basketball and other ball sports, typically reaching peak blood concentration one hour after ingestion [2,4]. While a review by Tan et al. (2022) [4] suggested caffeine supplementation improves vertical jump height and sprinting speed with a low-to-moderate effect size, there is no evidence about its influence on shooting accuracy. However, the slow absorption rate of caffeine through the gastrointestinal tract can led to fluid overload or gastrointestinal discomfort [9].

Caffeinated chewing gum offers a faster absorption rate compared to caffeine capsules, due to buccal mucosa absorption [10]. A study by Morris et al. (2019) [11] investigated the impact of chewing duration on caffeine release from gum and its pharmacokinetics. Participants chewed 100 mg of caffeine gum for 2, 5, and 10 min. The results demonstrated that chewing for 5 min achieved an 85% caffeine absorption rate, while chewing for 10 min resulted in almost complete absorption. Caffeine absorption likely occurs through both oral mucosa during chewing and the gastrointestinal tract upon ingestion. The highest blood caffeine concentration was observed after 15 min of chewing caffeinated gum [11]. From this research, the caffeine in caffeinated chewing gum can be absorbed as effectively as that in traditional energy drinks or coffee, and can reduce water absorption and urination, allowing athletes to control their water intake much easier during competitions and training. Regarding exercise performance, chewing gum containing 100–300 mg of caffeine 5–15 min before exercise can enhance aerobic capacity, reduce sprint speed decline, increase vertical jump height, and improve explosive power [5,12,13,14]. In a study by Chen et al. (2023) [15], it was found that chewing caffeinated gum with only 2.75 ± 0.53 mg/kg of caffeine was effective for increasing the peak power of lower extremity contractions [15]. The faster caffeine absorption rate may be responsible for caffeine’s effect on strength performance, but the associated physiological mechanisms remain unclear. Also, the impact on basketball-specific fitness and technical skills remains unclear.

While caffeine supplementation has demonstrated efficacy for enhancing basketball physical performance indicators, the effect of chewing caffeine gum on basketball-specific athletic ability remains unclear, particularly at relatively low dosages (3 mg/kg). Furthermore, flywheel inertial resistance exercise devices have established reliability for explosive power testing [16]. Better explosive power output has been found to have a significant positive correlation with sprint speed and vertical jump height. However, it remains unknown whether chewing caffeinated gum significantly enhances energy output during flywheel inertial resistance exercise and, subsequently, translates into enhanced basketball-specific athletic performance. Therefore, this study aimed to investigate the effects of chewing gum containing a relatively low dose of caffeine on basketball-specific exercise performance.

## 2. Materials and Methods

### 2.1. Study Design

**Figure 1:**
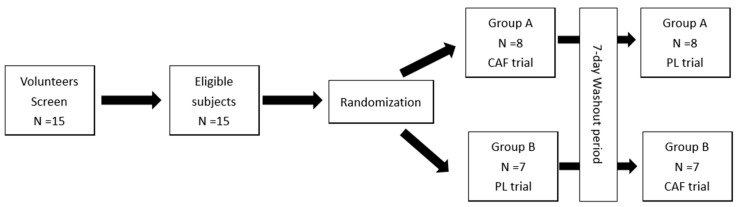
CONSORT diagram and study design.

A double-blind, repeated-measure crossover design was employed to investigate the effects of caffeine on basketball performance (Figure 1). The participants were randomly assigned to chew either a 3 mg/kg caffeinated gum (CAF) or a placebo gum (PL), followed by a seven-day washout period before crossing over to the other trial. Prior to the formal experiment, all the participants completed one or two familiarization tests to ensure proper test execution. Free throw accuracy was designated as the primary outcome measure, and the secondary outcomes were basketball-specific physiological tests. This study was conducted from 29 May 2023 to 8 September 2023. This is the out-of-season training period for players.

### 2.2. Participants

Fifteen healthy, adult male basketball players (age: 20.9 ± 1.0 years; height: 180.9 ± 5.4 cm; mass: 77.2 ± 7.5 kg; training age: 8.2 ± 0.3 years) were enrolled in this study. The inclusion criteria were as follows: (a) 6 years of professional basketball training with at least a top-8 national ranking; (b) 6 months of continuous training; (c) full recovery (at least 3 months) from sports injuries, such as strains and sprains. The exclusion criteria were as follows: (a) non-specialized basketball players; (b) not having trained regularly for the past 6 months; (c) not fully recovered from a sports injury (less than 3 months), or having a history of epilepsy, hypertension, hyperlipidemia, heart disease, arthritis, osteoporosis, brain injury, or a history of caffeine allergy; (d) participants with previous caffeine allergy responses. All the participants provided written informed consent after being fully informed about the potential risks involved in the experiment. A signed consent form was obtained from each participant. The order of the participants was determined through computerized randomization software (Excel Office 365, Microsoft, Washington, DC, USA). Two weeks before the formal experiment, the participants were asked to minimize their daily caffeine intake to less than 80 mg. This study received approval from the Institutional Review Board of Jen-Ai Hospital-Dali Branch (111-83) and was registered with ClinicalTrials.gov (Approval date: 4 August 2023; ID “NCT06016985”; https://register.clinicaltrials.gov, accessed on 19 March 2024). All the data were collected at the indoor stadium. This study was conducted following the Declaration of Helsinki.

### 2.3. Protocol

#### Pretests

Two pretests were conducted before the formal experiment. The pretests involved familiarization with the experimental apparatus, practicing the movements, and testing with different inertia loads. The load resistance was 0.010, 0.025, 0.050, or 0.075 kg, and the participants’ peak power was used as the experimental load resistance. The pretests were separated by 7 days, and the first phase of the formal experiment and the last pretest were also separated by 7 days to allow for adequate recovery of muscle performance. Similar pretesting procedures have been published in a past study [15].

The participants used an Exxentric inertial resistance training machine (Exxentric kbox 4 Pro, Stockholm, Sweden) to complete the harness squat movement test. With the maximum inertial resistance measured during the pretest being used as the resistance load during the experiment, the participants performed a maximum inertial resistance exercise comprising five sets of five reps, with a 3 min rest between. The participants were asked to perform all movements using their maximum strength as powerfully as possible. Verbal encouragement was also used to motivate the participants during the tests.

The second pretest involved the same procedure as the first pretest, but with the participants using the load that resulted in the highest power output identified during the first pretest; the pretests were conducted at least 48 h apart. Before each test, the participants underwent a standardized warm-up supervised by the individual conducting the experiment, which included riding on a stationary bicycle with a speedometer for 5 min (at a manageable intensity, that is, in the 10–12 range on the perceived exertion scale, with the full range being 6–20), a joint range-of-motion warm-up, and dynamic stretching for 3 min. Before the warm-up, the scale was explained. Twenty-four hours before the pretests, the participants were asked to avoid all caffeinated foods and beverages. The participants were also asked to reduce lower extremity exercise two days prior to the tests.

### 2.4. Experimental Procedure

All the participants completed the primary test 1–2 times prior to the formal experiment to familiarize themselves with the testing procedures. On the day of the main experiment, the participants were provided with standardized meals (breakfast and lunch) at designated times (8:00 a.m. and 12:00 p.m.) to control for pretest dietary variations that might influence performance. The meals were designed to provide a consistent macronutrient profile (11.9 ± 2.4% protein, 41.6 ± 16.7% carbohydrates, 26.8 ± 6.1% lipids) and energy intake (1353.6 ± 135.4 kcal). To control for the potential confounding effects of habitual caffeine intake, the participants were instructed to abstain from caffeinated beverages and foods for two weeks prior to the experiment. Prior to the experiment, we used a 3-day dietary record to determine that the average caffeine intake of the participants was 74.0 ± 24.2 mg.

Each experimental trial started at 3:00 p.m. Upon arrival at the court, the participants took a 10 min period of quiet rest. During the rest period, the participants in both the caffeine (CAF) and placebo (PL) trials chewed gum for 10 min. The CAF gum contained 3 mg/kg of caffeine, while the PL gum was non-caffeinated. The chewing time was based on a prior hemodynamic study demonstrating complete caffeine absorption from the gum within this timeframe. After chewing, the participants spat the gum out and rested quietly for 15 min. This rest period aligned with previous studies suggesting that chewing caffeine gum for 15 min before exercise optimized blood concentration levels [11], and enhanced performance in activities such as sprint speed, vertical jump height, and explosive power [5,12,13].

After resting, the participants engaged in a standardized dynamic warm-up routine, which included 15 m sprints, four times in total, followed by ten squat jumps. This warm-up typically lasted 10–15 min. Subsequently, the participants transitioned to a free throw shooting program to familiarize themselves with the shooting environment. The main testing then commenced in a predetermined sequence, designed to be completed within 45 min. The testing program consisted of a stationary free throw shooting test, countermovement jump, *t*-test, 20 m linear sprint test, squat in the flywheel device, and running-based anaerobic sprint test.

### 2.5. Outcome Measure

For the free throw accuracy test, the participants completed 3 sets of 10 free throw shots with a 1 min break [17]. The researchers confirmed that the participants did not violate the penalty line during the entire examination, and the average goal percentage of the free throw accuracy test was recorded.

The countermovement jump test was assessed using a jumping mat (Fusion Sport, Coopers Plains, QLD, Australia). Each participant stood on the jumping mat and maintained a still position, jumping with his hands on his hips without swinging. After hearing the tester’s instruction, each participant quickly performed a squat with their knees flexed in a 90-degree angle and then jumped vertically as soon as possible, allowing the feet to return to the jumping mat at the same time as a cushion. Three jumps were performed, and the average jump height was analyzed.

The T-shaped agility test is a standard method for assessing agility [18]. Four cone markers were set up in a T shape, and a timing light gate (Witty, Microgate, Bolzano, Italy) with an accuracy of 0.01 s was positioned at the starting position. The participants initialized the test with a maximal sprint 10 yards from the first cone marker to the second, followed by a left side step for 5 yards to the third cone marker. They then performed a right side step for 10 yards to the fourth cone marker, and immediately returned back to the second cone marker with a left side step for 5 yards. Finally, the participants sprinted back to the first cone marker to conclude the test. During the test, the participants had to touch each cone marker with their hand before moving to the next one.

The 20 m segmented sprint test was conducted using a timing grating (Witty, Microgate, Bolzano, Italy) placed at the starting point and at 10 m and 20 m. The participants sprinted 20 m forward as fast as they could, two times in total, with a 2 min break each time. The average completion time was recorded.

The flywheel inertial resistance exercise test consists of 3 sets of 5 repetitions on an inertial resistance trainer (K-Box 4, Exxentric, Stockholm, Sweden) using a harness squat [19]. Each participant, wearing an orthopedic undershirt, stepped onto the inertial resistance machine, cantered himself on the device, set the counterweights on the machine’s underside, and fastened them. After the test, the participants were instructed to squat as hard as possible until their thighs were parallel to the ground. They completed five repetitions of the squat, and the average power, peak concentric force, and peak eccentric power during the squats were recorded.

The anaerobic sprint test employed six maximal-effort 35 m sprints separated by 10 s rest intervals to measure peak power, mean power, minimum power, and fatigue index. It has been shown to have adequate reliability [20] for the accurate measurement of the fatigue index. Two timing gates were positioned as follows: one at the starting point and another at the 35 m endpoint, to ensure a sufficient buffer zone for the 10 s rest period. When the participants were ready, they stayed still and completed six maximum-effort 35 m sprints with a 10 s rest between sprints. The completion time for each sprint was recorded for subsequent analysis.

### 2.6. Caffeine and Placebo Gum

The caffeinated chewing gum used in this study (Military Energy Gum, Arctic Mint flavor; Stay Alert, Chicago, IL, USA) has been used in previous studies [15,21]. Each piece contained 100 mg of caffeine with a 5 g gum base. Different weights of gum were given to the participants according to the relative dosage. A commercially available blue mint gum served as the placebo. To achieve a target dose of 3 mg caffeine per kilogram of body weight, and to maintain the double-blind design, all the chewing gums were mashed, ground, homogenized, and reshaped after incorporating 0.3 g of peppermint flavoring powder. This ensured an indistinguishable color, appearance, taste, weight, and size between the gum types. All the chewing gums were prepared by specialized personnel and given to the on-site testers after numbering. After the gum was chewed, a questionnaire was given to the participants to confirm whether they could tell the difference between the two chewing gums. In the CAF trial, 8 out of 14 participants (57%) incorrectly identified the presence of caffeine. Similarly, in the PL trial, 6 out of 14 participants (42%) were unable to correctly identify the placebo.

### 2.7. Sample Size Calculation

We employed G*Power 3.1.9.6 [22] to determine the required sample size based on data from a previous investigation [15]. A prior study suggested that chewing caffeinated gum could significantly increase the eccentric peak force during a Romanian deadlifting exercise [15]. An analysis of the data using a paired *t*-test revealed that a total sample size of approximately 15 participants was necessary to achieve 80% power to detect significant variances in postprandial plasma glucose levels due to chewing among healthy participants. This calculation assumed an alpha level of 0.05 and a correlation coefficient of 0.55. The determination of the sample size was designed to detect an effect size of 0.5 (Cohen’s d) using a paired *t*-test for the two trial conditions. Following these calculations, 15 participants were enrolled in this study.

### 2.8. Statistical Analysis

All the data are presented as means ± standard deviations. The Shapiro–Wilk test was used to assess the normality of the data distribution. The stationary free throw shooting test, countermovement jump, *t*-test, 20 m segmented dash test, squat in the flywheel device, and running-based anaerobic sprint test were employed to analyze the through paired *t*-tests. Effect sizes were calculated using Cohen’s d to quantify the magnitude of the observed effects. The power value of each data point was conducted using G*Power 3.1.9.6 software [22]. All the data were calculated using SPSS (version 20, Chicago, IL, USA), and the significance level was *p* < 0.05.

## 3. Results

### 3.1. The Free Throw Accuracy Test

**Figure 2:**
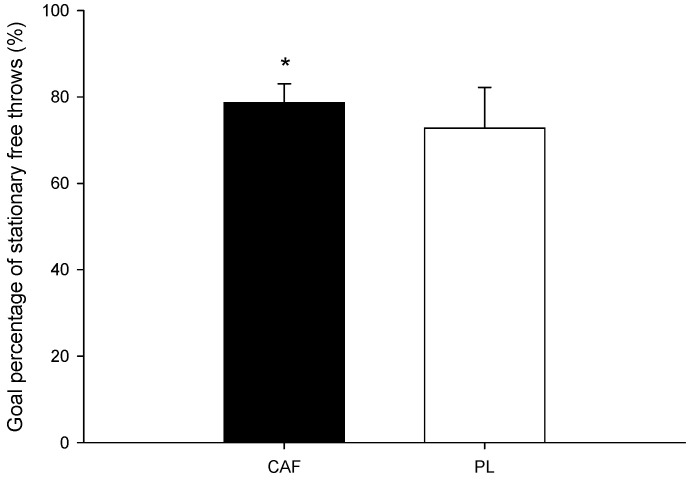
The goal percentage of stationary free throws. * The CAF value was significantly higher than that for the PL.

For the free throw accuracy test, the average goal percentage across the three sets (Figure 2) was significantly higher for the CAF trial compared to the PL trial (CAF: 79.0 ± 4.31%; PL: 73.0 ± 9.16%; *p* = 0.012; Cohen’s *d* = 0.94; Power = 0.90).

### 3.2. CMJ and Agility Testing

**Figure 3:**
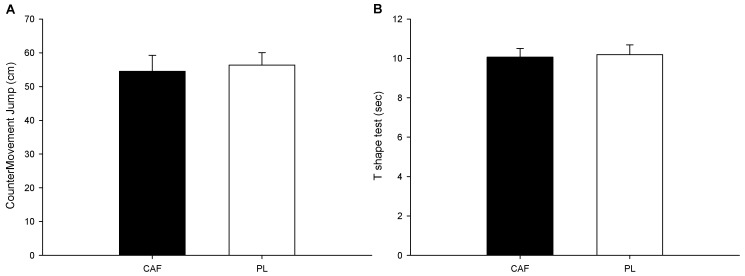
CMJ and agility testing. The jump height (**A**) and the T-shape agility test (**B**).

The average CMJ jump heights are presented in Figure 3A. The statistics show no significant difference between the two trials (*p* = 0.147). The *t*-shaped agility test (Figure 3B) shows no significant difference between the two trials (*p* = 0.571).

### 3.3. The 20 m Segmented Sprint Test

**Figure 4:**
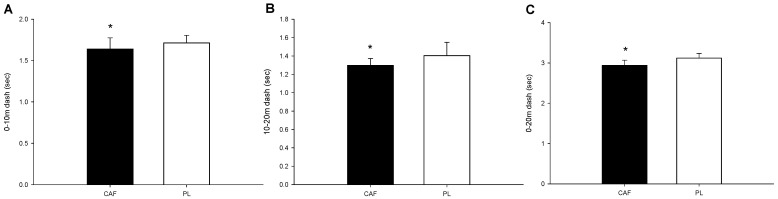
The 20 m segmented sprint test. The completion times for 0–10 m (**A**), 10–20 m (**B**), and 0–20 m (**C**). * The CAF values were significantly lower than those for the PL.

The completion times for the 0–10 m (*p* = 0.045; Cohen’s *d* = 0.94; Power = 0.95; Figure 4A), 10–20 m (*p* = 0.019; Cohen’s *d* = 0.70; Power = 0.89; Figure 4B), and 0–20 m (*p* < 0.001; Cohen’s *d* = 1.8; Power = 0.99; Figure 4C) were all significantly faster for the CAF trial compared to the PL trial.

### 3.4. The Flywheel Inertial Resistance Exercise Test

**Figure 5:**
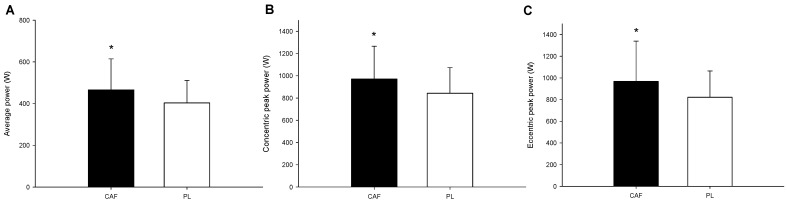
The flywheel inertial resistance exercise test. The average power (**A**), peak concentric power (**B**), and peak eccentric power (**C**) for the harness squat in the flywheel inertial resistance device. * The CAF values were significantly higher than those for the PL.

The harness squat performance in the flywheel inertial resistance device showed that the average power (*p* = 0.012; Cohen’s *d* = 0.41; Power = 0.80; Figure 5A), peak concentric power (*p* = 0.013; Cohen’s *d* = 0.48; Power = 0.81; Figure 5B), and peak eccentric power (*p* = 0.028; Cohen’s *d* = 0.45; Power = 0.80; Figure 5C) were all significantly lower for the CAF trial compared to the PL trial.

### 3.5. Running Anaerobic Sprint Test

Caffeinated chewing gum significantly improved the running anaerobic sprint test (RAST) results between the CAF and PL trials (Table 1). The statistical analysis reveals significantly higher minimum power (*p* = 0.008) and minimum power per weight (*p* = 0.011) for the CAF trial compared to the PL trial. The fatigue index data show a significantly lower value (*p* = 0.009) for the CAF trial compared to the PL trial, with a large effect size (Cohen’s *d* = 1.00) and actual power (0.96).

## 4. Discussion

Basketball performance can be explored in terms of shooting and physical performance indicators. For shooting ability, it is clear from this study that chewing gum containing 3 mg/kg of caffeine significantly improved the free throw accuracy with a large effect size, but did not enhance the CMJ jump height or the T-shaped agility test performance. However, it did improve the physical performance indicators, including the 20 m sprinting ability, squat strength, and reduced fatigue index with moderate-to-large benefits. Based on these data, chewing caffeinated gum before basketball training or competition is a way to improve basketball shooting accuracy and physical performance indicators.

This study found that after chewing caffeine gum, there was a significant increase in free throw accuracy with a high effect size. This may be related to the fact that caffeine intake can effectively improve mental focus and coordination. Both of these indicators are possible causes of free throw accuracy. A clinical study published in 2002 investigated the effects of caffeine on exercise performance on a low-intensity task. Thirty-one university students were randomized into a single-blind caffeine trial and a placebo trial with a caffeine dosage of 5 mg/kg, and were given either the caffeine or the placebo intervention, followed by a 30 min hand–eye coordination test. Hand–eye coordination was tested 30 min later. The results showed that caffeine intake effectively improved hand–eye coordination [23]. However, a past systematic review concluded that caffeine supplementation did not improve shooting ability [4]. In contrast to these past experiments, the present study, in which caffeine at a lower dosage (3 mg/kg) was administered via caffeinated chewing gum, found that the CAF trial significantly increased free throw shooting accuracy with a high degree of effect size. It seems that chewing caffeine gum can achieve the maximum concentration of caffeine in the bloodstream more quickly, allowing caffeine to have a better effect on hand–eye coordination and cognitive ability. Moreover, the standard deviation in the caffeine trial was smaller than that in the placebo trial. This data may also suggest that caffeine stabilized the participants’ hand–eye coordination or body control. To understand whether this mechanism is correct, future studies are expected to investigate whether different absorption rates of caffeine affect cortical activation by EEG and reveal alpha peak frequency [24], and by using cognitive function tests.

Another important finding of this study is that chewing caffeine gum can effectively increase sprinting speeds for 20 m, whether it is the beginning 0–10 m or the second half of the 10–20 m distance. This suggests that caffeine gum can effectively shorten running time. Short-distance sprinting ability is mainly related to lower-body strength and power [25,26]. Our results from the harness squat performance in the flywheel inertial resistance device showed significantly higher peak concentric and eccentric power for the CAF trial compared to the PL trial. This increased power output may be the primary mechanism by which caffeine gum improves sprinting speed. However, a past systematic review and meta-analysis has demonstrated inconsistent results regarding the effects of caffeine supplementation on lower-extremity strength [27]. Interestingly, a recent systematic review and meta-analysis by Barreto et al. (2023) found that chewing caffeine gum 15 min before exercise effectively increases muscle strength and explosive power [13]. While more research is needed to explore the effects of higher caffeine bioavailability on sprint performance, this study demonstrates that chewing caffeine gum containing 3 mg/kg of caffeine 15 min before exercise can improve sprinting speed.

This study demonstrates that chewing caffeinated gum effectively reduces fatigue during sprinting, aligning with previous studies on caffeine supplementation via capsules [28]. To assess repetitive sprinting ability, researchers often employ tests such as the Wingate test or repeated maximal speed sprints. Schneiker et al. (2006) [29] conducted a randomized, crossover, double-blind experimental design study with ten healthy adult males. The participants received either 6 mg/kg of caffeine or a placebo 60 min before exercise. They then performed 18 sprints of 4 s each with a 2 min rest period. The study found caffeine significantly increased sprint work and the mean peak power [29]. Similarly, Paton et al. (2010) [30] examined the effects of caffeine gum on nine trained male cyclists. The participants chewed 240 mg of caffeine gum and performed a sprint test involving 30 s sprints repeated five times per round, for a total of four rounds. The study showed that caffeine gum significantly reduced the decline in power output during high-intensity repetitive sprinting and effectively reduced perceived fatigue [30]. Evans et al. (2018) [31] investigated the impact of caffeine gum on non-habitual caffeine consumers. Ten adult males chewed either 200 mg of caffeinated gum or a caffeine-free placebo before performing ten 40 m sprints with 30 s rest intervals. Their findings revealed that chewing caffeine gum significantly increased speed loss during repeated sprints among the participants who did not normally consume caffeine [31]. However, their study did not assess the fatigue index. Therefore, the present study provides new evidence. We find that using caffeinated chewing gum effectively reduces the fatigue index during repetitive sprinting, as measured by the running anaerobic sprint test.

The data in this study show that chewing caffeinated gum did not significantly affect CMJ jump height or agility performance. The stretch–shortening cycle and stretch reflex may be necessary for influencing CMJ jump height and agility [32,33]. In the present study, the peak concentric power was very similar to the peak eccentric power. This may suggest that the participants did not utilize the stretch reflex effectively during jumping, potentially explaining the lack of improvement in jump height and agility. On the other hand, this study was conducted during the off-season. The off-season may have an impact on a player’s body composition, aerobic capacity, and cognitive ability [34,35]. Whether these changes will affect the results of this study remains unclear. Future studies could examine the effects of caffeinated gum chewing on the stretch reflex in athletes at different stages of preparation.

### Strengths and Limitations

The strengths of this study include demonstrating that caffeine supplementation via chewing gum, even at a lower dose (3 mg/kg), can significantly enhance the sport-specific physiological and technical performance of trained basketball players. We not only confirmed the effect size of the data but also calculated the actual power value to strengthen data credibility. A review by the International Society for Nutrition [5] reported a moderate-to-low effect size on the strength and sprinting performance of athletes given 3–6 mg/kg caffeine. Our study employed a relatively low dose (3 mg/kg), and suggested that the rapid absorption of caffeine from chewing gum may be effective for enhancing the moderate-to-high effects observed in 20 m sprints and squat strength.

Limitations exist despite our efforts to optimize the experiment. First, we did not measure blood caffeine concentration, leaving the exact blood caffeine value unknown. However, a previous study using the same brand of caffeinated chewing gum showed the peak blood caffeine concentration after a 10 min chew followed by a 15 min rest (Morris et al., 2019) [11]. Therefore, our chewing duration and rest periods should have been sufficient for peak blood caffeine levels. Second, we did not measure heart rate variability or brainwave changes, hindering our ability to pinpoint the exact mechanism by which caffeinated gum enhances athletic performance. While our data lack direct evidence of the specific mechanism, this finding provides a new direction for future research. This could involve examining the effects of high caffeine absorption from caffeine chewing gum on sympathetic nerves or brainwaves to identify the potential mechanisms.

## 5. Conclusions

This study found that chewing caffeine gum containing 3 mg/kg of caffeine before exercise may lead to moderate-to-large improvements in key performance attributes for professionally trained basketball players. This finding may indicate that caffeine gum has a faster absorption rate and bioavailability of caffeine from chewing gum. Future research could investigate the caffeine concentration in saliva or blood after consuming caffeinated chewing gum to confirm the relationship between faster caffeine absorption and exercise performance. Moreover, to explore the potential mechanisms underlying these observations, future studies are expected to investigate whether different caffeine absorption rates influence cortical activation by EEG, alpha peak frequency, and cognitive function tests.

## Figures and Tables

**Table 1:** Results of running anaerobic sprint test (RAST) between CAF trial and PL trial.

	CAF	PL	*p* Value	Cohen’s *d*
Peak power (W)	1354.86 ± 44.2	1326.70 ± 75.0	0.328	0.46
Mini power (W)	1234.44 ± 75.7	1153.90 ± 35.9	0.008 *	1.35
Peak power per weight (W*kg^−1^)	17.92 ± 1.9	17.53 ± 1.8	0.323	0.21
Mini power per weight (W*kg^−1^)	16.30 ± 2.1	15.26 ± 1.6	0.011 *	0.53
Fatigue index (%)	3.60 ± 1.6	5.21 ± 1.6	0.009 *	1.00

Values are mean ± SD, *n* = 15. CAF, caffeine trial; PL, placebo trial. * CAF values were significantly different than those for the PL.

## Data Availability

All relevant materials are presented in the present manuscript.
